# Synthesis of core–shell Ce-modified mixed metal oxides derived from P123-templated layered double hydroxides†

**DOI:** 10.1039/d1ra00227a

**Published:** 2021-02-23

**Authors:** Kaijun Wang, Qifan Mao, Weimin Fei, Lingxin Kong, Xiaoyan Cao, Zhenggui Gu

**Affiliations:** Jiangsu Provincial Key Laboratory of Materials Cycling and Pollution Control, Nanjing Normal Univesity Nanjing Jiangsu 210023 China 07160@njnu.edu.cn

## Abstract

Layered double hydroxides are a promising platform material which can be combined with a variety of active species based on their characteristic features. Silicon@P123-templated Ce-doped layered double hydroxide (SiO_2_@CeMgAl-LDH(P123)) composites were synthesized *via* a facile *in situ* co-precipitation method, and characterized by TEM, X-ray diffraction, FTIR, XPS, CO_2_-, *etc.* in detail. Meanwhile, the calcined powder (SiO_2_@CeMgAl-LDO(P123)) possessed an excellent core–shell structure and a high surface area inherited from the LDH structure, which led to an outstanding catalytic activity (99.7% conversion of propylene oxide, 92.4% selectivity of propylene glycol methyl ether) under mild reaction conditions (120 °C). Cerium oxide provides a large number of oxygen vacancies and significantly improves the medium basic strength of the material, which facilitates the selective ring-opening of PO. Furthermore, the introduction and removal of P123 make the cerium oxide uniformly dispersed on the LDH layers, providing more reaction sites for the reaction of methanol and propylene oxide. The core–shell structure prepared by the *in situ* co-precipitation method could solve the shortcomings of agglomeration of layered double hydroxides and prolong the catalytic life evidently.

## Abstract 2

A shell of P123-templated CeMgAl-LDO was distributed in transverse and longitudinal directions on spheres of SiO_2_. The composites displayed high catalytic activity in the synthesis of propylene glycol methyl ether.

## Graphical abstract

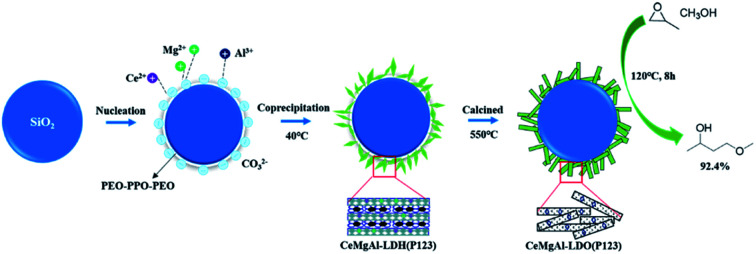

## Introduction

Low toxicity, high efficiency, and a stable solvent are indispensable features in a green chemical process. Propylene glycol ethers are important industrial derivatives of epoxides by etherification. Their molecular structure has two strong soluble functional groups: a hydroxyl group and ether bond group. The hydroxyl group is hydrophilic while the ether bond is lipophilic, so this kind of material is also called a “universal solvent”.^1–3^ Among the many synthetic routes of propylene glycol ether, the propylene oxide method has a high atom utilization rate and low operation risk, so it has become the only green synthesis route to realize industrialization.^4^ However, due to the steric hindrance effect of propylene oxide, different products will be produced under the action of acid and base in the reaction process of propylene oxide and lower fatty alcohol. The primary hydroxy ether is the main product in the presence of acid-type catalysts. At the same time, basic catalysts favor the formation of secondary hydroxy ether. Some studies have shown that secondary alcohols have lower reproduction and developmental toxicity than primary alcohols. Therefore, it is of great significance to improve the selectivity of secondary alcohols.

Although traditional solid base catalysts such as NaOH and KOH have good catalytic activity in similar alkoxylation reactions, such homogeneous and highly corrosive substances do great harm to industrial equipment and increase the difficulty of product refining. Recent researches of alcohol ether synthesis took ionic liquids^5^ and zeolitic imidazolate framework (ZIF-8)^6^ as important research objects. These new types of catalysts can achieve a high PO conversion rate with a relatively small amount of use, but they become a stumbling block in the product purification process. When used in the actual production process, the above catalysts are difficult to achieve the expected results. Heterogeneous catalysts are widely used in the synthesis of chemical products. They reduce the corrosion of equipment and the difficulty of product refining. Moreover, most of the heterogeneous catalysts have the advantages of simple preparation, controllable morphology and long catalytic life. It is the main trend of the development of chemical industry and one of the important ways to realize green chemical process.

In recent ten years, there have been a lot of reports on the synthesis of alcohol ether with heterogeneous solid base catalysts. Timofeeva prepared Al-pillared interlayered clays (Al-PILCs)^7^ and Zr,Al-pillared clays through the intercalation of Al- and Zr-polyoxocations into layered aluminosilicates with various textural properties and chemical compositions^8^ to explore the synthesis process of alcohol ethers. The catalytic performance of these materials was investigated in the synthesis of PGME. 0.4% Zr Al-MM_NO_3_^−^_^K^ got a certain catalytic activity at 60 °C that conversion of PO was 71% and selectivity towards to PGME was 66%. The research found that the increase of Zr content was beneficial to decrease the selectivity of PGME, which was the result of a change in the nature of acid–base sites. The highest conversion and selectivity toward PGME were observed in the presence of materials with medium-strength basic sites, because high-strength basic sites can lead to a strong stabilization of methoxide and propylene-like species on the surface of the solid. Liu^9^ combined KF with La_2_O_3_-SBA-15 at low temperature to form ordered microstructure on the solid surface and generate superbase sites. The conversion of propylene oxide was 93%, and the selectivity of 1-methoxy-2-propanol was 93%. For the solid base catalyst used in this system, the higher specific surface area and the medium strong base strength can promote the yield of PGME obviously.^10^ Recently, for another piece of research, Timofeeva^11^ found that the catalytic properties of AM-4 could be adjusted by treatment with 0.0625–0.25 M HNO_3_. Increasing the concentration of HNO_3_ led to a decrease in basicity, which played a critical role in the reaction rate and the selectivity towards PGME. Their activity depended on the type of active sites, which is controlled by acid concentration. In a word, the more active sites with moderate and strong base strength and larger specific surface area are more conducive to the formation of secondary hydroxy alcohol ether.

The LDH structure is based on M(OH)_6_ octahedral units sharing edges in order to build M(OH)_2_ brucite-like layer.^12^ LDH plays an important role in the field of industrial catalysis. This is not only its special layer structure and ion exchange properties, but also it can be used as a carrier or an active component. This feature greatly expands the possibility of material topography modification. Therefore, in recent years, LDH-based multi-level structural composite materials have become the focus of attention.^13–16^ The metal components in the hydrotalcite laminate can be flexibly adjusted, so a wide variety of hydrotalcite-like catalysts can be designed for different reaction systems.^17–19^ Cerium is a rare earth element with the highest abundance. The crystal of CeO_2_ has the structure of cubic fluorite with primary characteristic surfaces of (111), (110) and (100). Due to the differences in the packing of atoms in bulk structure and certainly at surface, the interaction forces between the surfaces and the reactants would be different.^20,21^ The specific surface area, dispersion of active species, oxygen cavity defect and lattice oxygen fluidity of CeO_2_ can be changed by metal doped CeO_2_ and the interaction between metal and CeO_2_. Therefore, cerium based composite metal oxides have been widely used in the field of catalysis.^22–24^ Recently, incorporation of cerium oxide based nanostructured materials into layered double hydroxide to form composite material is another burning topic of research interest.^25,26^ Core–shell nanoparticles (CSNs) are a class of nanostructured materials that have recently received increased attention owing to their interesting properties and broad range of applications in catalysis, biology, materials chemistry and sensors.^27^

In our previous work, we have explored how to increase the specific surface area of the catalyst while ensuring the basic strength. We proved that the core–shell functional materials with specific morphology can be prepared by certain methods.^28^ We found that MgAl-LDH supported on SiO_2_ surface by *in situ* coprecipitation method can form core–shell materials with composite shell distribution in transverse and longitudinal directions. After calcination, the composite structure still maintains a certain integrity, and solves the problem that LDH is easy to agglomerate. At the same time, it shows ideal catalytic activity in fixed bed reactor.

**Fig. 1:**
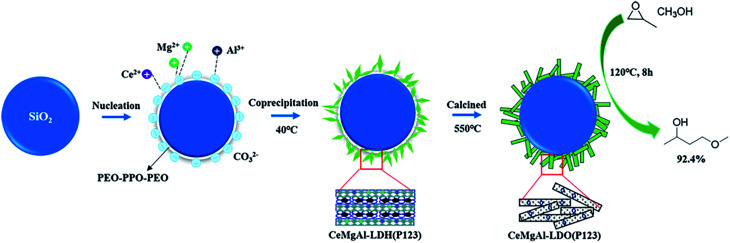
Schematic illustration of formation of SiO_2_@CeMgAl-LDO(P123) nanocomposites.

Taking into consideration the above discussion, we report herein a novel SiO_2_@CeMgAl-LDH nanocatalyst, which was easily fabricated by the *in situ* coprecipitation of the CeMgAl-LDH anchored on the surface of amorphous SiO_2_ sphere. The template agent P123 is introduced in the material and removed by calcination for further treatment. The obtained material (SiO_2_@CeMgAl-LDO(P123)) was fully characterized and its catalytic activity was evaluated in the synthesis of PGME (Fig. 1). The catalytic performances of the as-prepared samples were correlated with the observed structural evolution of the catalysts. More importantly, this work could provide some directions and perspectives for the preparation of stable core–shell structure catalysts.

## Results and discussion

### Structural and morphology characterization

**Fig. 2:**
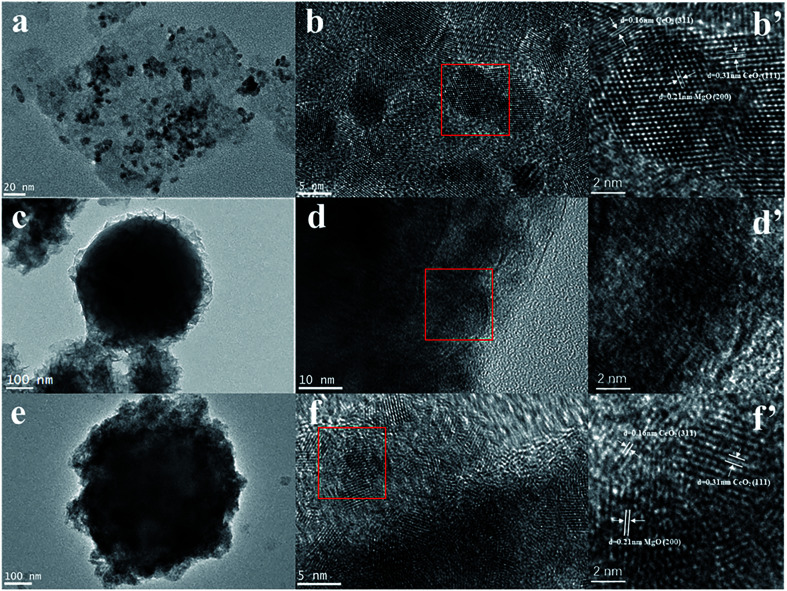
TEM images of samples (a, b and b′) CeMgAl-LDO, (c, d and d′) SiO_2_@CeMgAl-LDH(P123), (e, f and f′) SiO_2_@CeMgAl-LDO(P123).

The TEM images of the synthesized CeMgAl-LDO(P123), SiO_2_@CeMgAl-LDH(P123), and SiO_2_@CeMgAl-LDO(P123) are shown in Fig. 2. Fig. 2a (CeMgAl-LDO) shows a well-developed layered and platelet structure. Due to the introduction of P123 and its removal at a high temperature, it can be seen that small CeO_2_ grains are scattered on the LDO surface, and their average particle sizes are 4 and 4.5 nm, respectively. The LDH platelets grow vertically and randomly on the surface of the sphere SiO_2_ to form a hierarchical layer with thickness of around 50 nm (Fig. 2c). After calcination at 550 °C, the shell layer is transformed into multi-component metallic oxides, but the core–shell structure still maintained, indicating that the composite prepared by coprecipitation method have good structural stability (Fig. 2e). Through the further analysis of the shell morphology, different kinds of crystal lattice can be observed. These correspond to the (111) and (311) planes of CeO_2_ and (200) planes of MgO, respectively. These are both found in the shell layer of CeMgAl-LDO and SiO_2_@CeMgAl-LDO(P123).

**Fig. 3:**
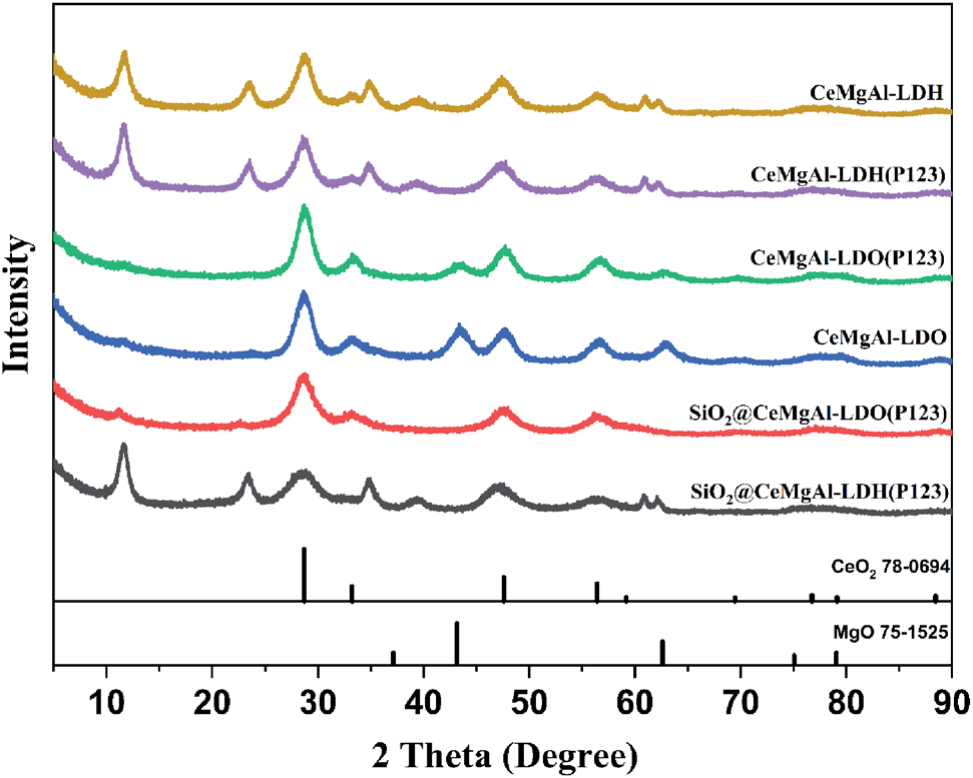
XRD patterns of CeMgAl-LDH, SiO_2_@CeMgAl-LDH, and calcined samples.

The crystal structure of the as-prepared materials was confirmed by XRD analysis (Fig. 3). The CeMgAl-LDH and CeMgAl-LDH(P123) samples exhibit the characteristic diffraction peak of LDHs, indicating that Ce-doping and P123 addition does not destroy the layered structure of LDHs. Ce prefers to be located in the interlayer gallery of LDH as agglomerated cerium oxide species due to its high ionic radius (0.102 nm). After calcination, CeMgAl-LDO is mainly composed of amorphous Al_2_O_3_, MgO and CeO_2_. Peaks at 28.7°, 33.2°, 47.7° and 56.6° are corresponding to the (111), (200), (220), (311) planes of Fluorite CeO_2_ (JCPDS PDF#78-0694). MgO may gradually enter the CeO_2_ lattice to form a new solid solution structure. Moreover, there is no obvious diffraction peak of heterophase species in the whole XRD spectrum of CeMgAl-LDO, which shows that the metal oxides prepared by nucleation crystallization isolation method have pure crystal phase composition. The XRD patterns of SiO_2_@CeMgAl-LDH(P123) and SiO_2_@CeMgAl-LDO(P123) both show an LDH/LDO-originated diffraction pattern which confirmed the formation of the LDH/LDO on the silica sphere. The intensity of LDH/LDO originated peaks decreased due to the coexistence of amorphous SiO_2_. It was reported that Si–O–Mg and Si–O–Al bonds on the SiO_2_ surface deeply affected reduction of the crystallite size not only in the stacking direction but also in the plane direction.^29^

**Fig. 4:**
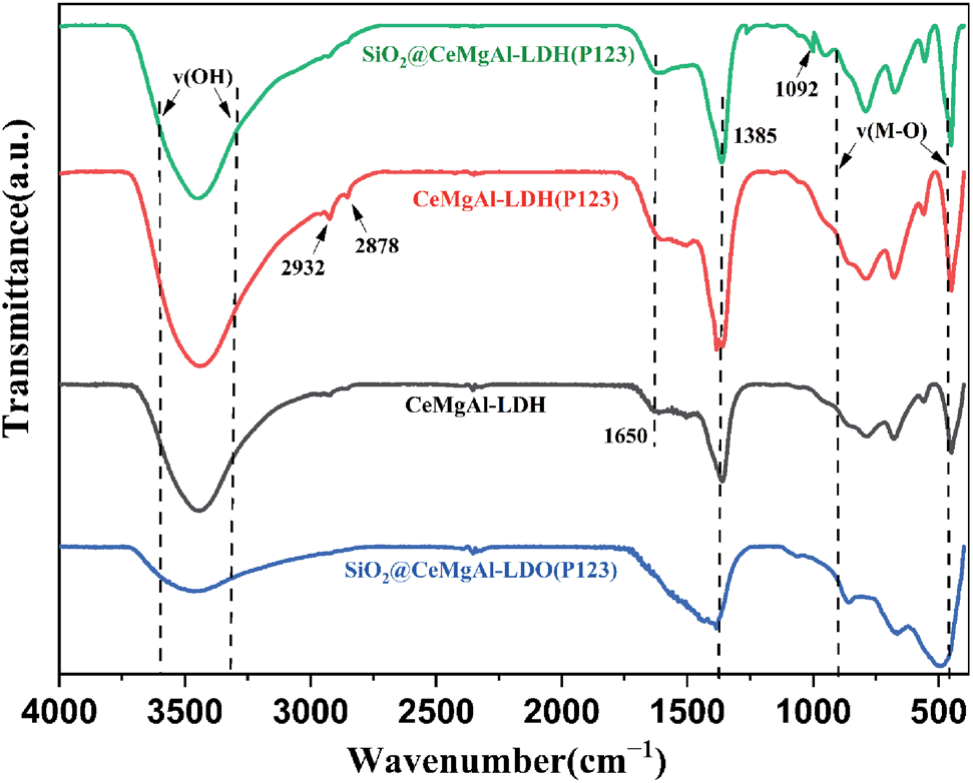
FT-IR profile of LDH, SiO_2_@CeMgAl-LDH, and calcined samples.

The FT-IR spectra are shown in Fig. 4. The peaks located in the 3000–3500 cm^−1^ region can be ascribed to the characteristic absorption bands of O–H attached to the metal cation bonds in the hydroxide layer and stretching vibration of interlayer water molecules. The width and relative strength of –OH in LDO decreased significantly due to hydroxyl removal caused by the calcination process. The bending vibration of interlayer water molecules are found at 1650 cm^−1^. Similar to the change in the –OH vibration peak, the absorption peak of LDO here basically disappears as water is removed between layers of the roasting process. A new peak appears at around 1092 cm^−1^ which could be supposed to the stretching vibrations of Si–O–Si and Si–O–H, indicating that SiO_2_ spheres are coated by the LDH platelets successfully in core–shell composites. The peak between 500 and 750 cm^−1^ can be assigned to the stretching vibrational mode of the Ce–O bonds.^30^ The peaks at 2932 and 2878 cm^−1^ are attributed to the alkyl stretching vibrations, both of which indicate the presence of P123 in as-synthesized materials.^31^ The FT-IR spectra indicated that the CeMgAl-LDHs/LDOs were involved on the surface of SiO_2_.

**Fig. 5:**
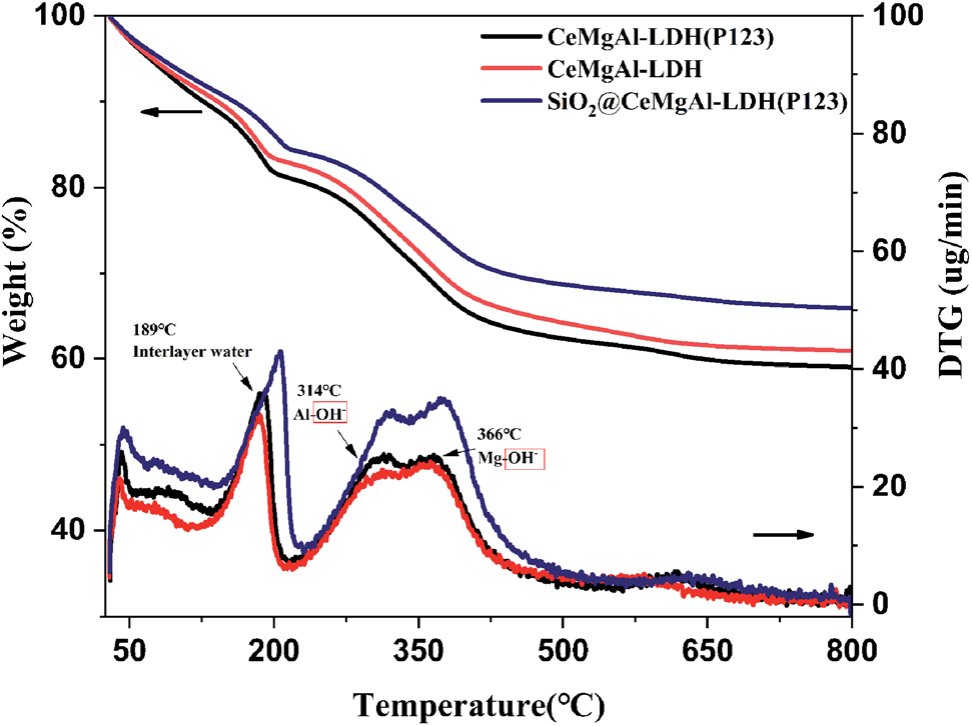
TG–DTA profile of samples.

It is known that calcined LDHs, layered double oxides (LDOs), are useful intermediates for functionalised LDHs and that their tuneable acid/base properties can be utilised in catalytic applications.^32^ The TGA-DTG curves of samples are shown in Fig. 5. Compared to Mg–Al LDH, all the samples show a similar thermal behavior as follow: (1) in the temperature range 70–190 °C, loosely held interlayer water is lost; (2) in the temperature range 190–280 °C, the OH– group bonded with Al^3+^ is lost; (3) in the temperature range 280–450 °C, the OH– group bonded with Mg^2+^ is lost; (4) the weight of the three composites tends to be stable after 500 °C. The introduction of P123 makes the three weightlessness temperatures shift to high temperature. This is due to the hydrogen bonding between P123 and OH^−^ or H_2_O, so higher energy requirements put forward in the process of chemical bond transformation.

**Fig. 6:**
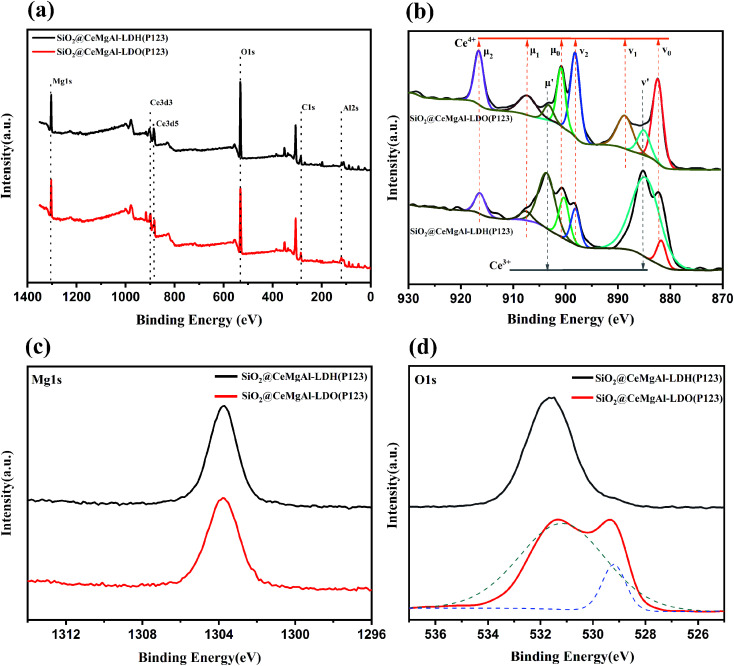
(a) The XPS survey and high resolution scans of samples, (b) the Ce 3d high resolution spectrum of SiO_2_@CeMgAl-LDH/LDO(P123), (c) the mg 1s high resolution spectrum of SiO_2_@CeMgAl-LDH/LDO(P123), (d) the O 1s high resolution spectrum of SiO_2_@CeMgAl-LDH/LDO(P123).

The X-ray photoelectron spectroscopy (XPS) was used to study the oxidation state of the surface elements in SiO_2_@CeMgAl-LDO(P123), and SiO_2_@CeMgAl-LDH(P123). The Ce 3d, Mg 1s, and O 1s regions of the spectra are shown in Fig. 6b–d. The binding energy peaks at 1301 and 117 eV can be attributed to Mg 1s and Al 2s, respectively. The HXPS spectra of Ce 3d before and after samples being calcined indicate the presence of the mixed valence Ce^3+^ and Ce^4+^ states. The main peaks of Ce^3+^ 3d_3/2_ and Ce^3+^ 3d_5/2_ are shown at the bending energy of 990 eV and 882 eV. One additional peak at 907.6 eV is also attributed to the Ce 3p3d_3/2_ states. The peaks of Ce 4p are found at the binding energies of 916.5 eV.^32–34^ It shows that cerium exists in similar form in both materials, but the relative content of some Ce^4+^ increases obviously after calcination. More importantly, it is beneficial for Ce^3+^ to lose an electron to be oxidized to Ce^4+^, improving the lattice oxygen defects and the redox transformation between Ce^3+^ and Ce^4+^.^35^ The O 1s binding energy peak appeared at 529 eV in SiO_2_@CeMgAl-LDO(P123) was assigned to Ce–O, peak at 531 eV with oxygen vacancies of Ce^3+^ in CeO_2_ and surface oxygen species in the layered structure of LDH/LDO.

**Fig. 7:**
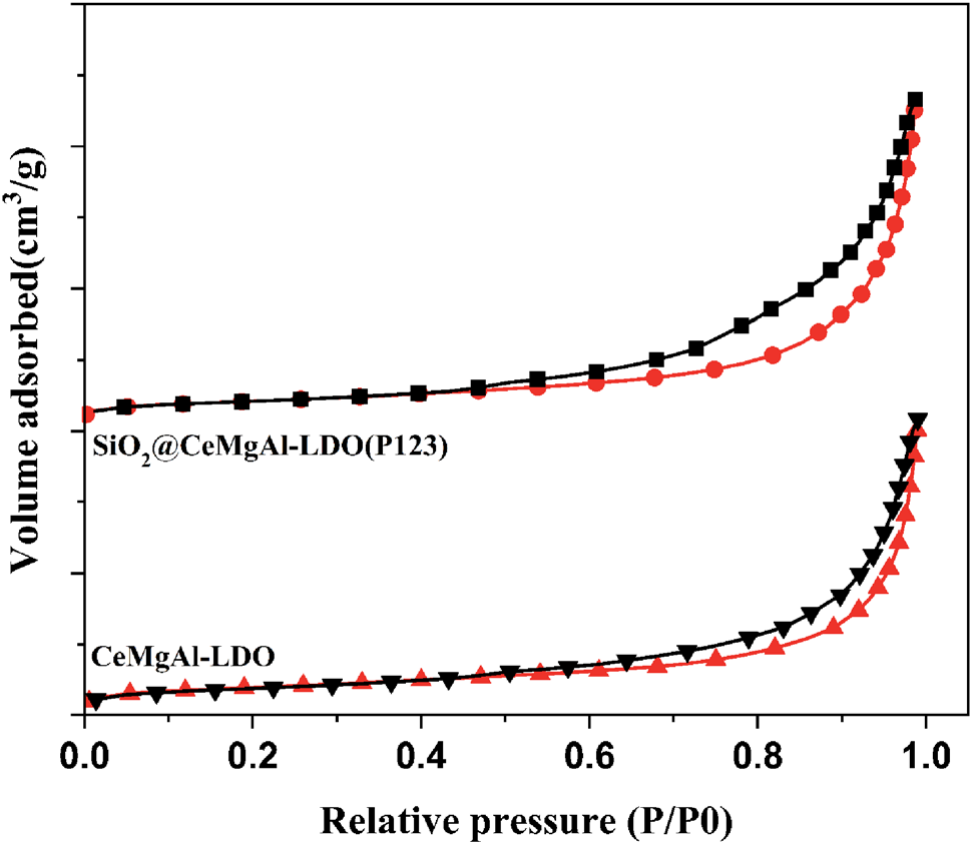
N_2_ adsorption/desorption isotherms of CeMgAl-LDO and SiO_2_@CeMgAl-LDO(P123).

The specific BET surface area and pore volumes of CeMgAl-LDO, and SiO_2_@CeMgAl-LDO(P123) are shown in Fig. 7, S2† and Table 1. It can be easily found that SiO_2_@CeMgAl-LDO(P123) showed much higher specific BET surface area (358.71 m^2^ g^−1^) and larger pore volumes (1.13 cm^3^ g^−1^) compared with CeMgAl-LDO (144.94 m^2^ g^−1^; 0.72 cm^3^ g^−1^). As can be seen from Fig. 7, nitrogen adsorption–desorption isotherms of CeMgAl-LDO, and SiO_2_@CeMgAl-LDO(P123) both exhibit intermediate isotherms between type II (absence of a plateau at high *P*/*P*_0_) and type IV (low N_2_ adsorption at low *P*/*P*_0_), with a hysteresis loop in the relative pressure range of 0.4 to 1.0, indicating the presence of capillary condensation in the mesopore structure. According to the IUPAC classification, interestingly, these two samples present a mixture hysteresis loop of H2 and H3 types with an abrupt closure at a relative pressure of 0.45 due to the tensile strength effect, suggesting the presence of slit-shaped and ink-bottle pores in the mesopore network associated with the aggregates of plate-like particles.^36^ In the adsorption desorption curve of SiO_2_@CeMgAl-LDO(P123), the hysteresis loop is larger, which shows that there are more complex hole structures. During the formation of the nuclear shell structure, the shell structure is relatively complex, with different forms such as horizontal fit, vertical growth and mixed construction. Due to the pore forming process of P123, the pore structure becomes more complex.

**Table 1:** BET surface and base sites of samples^a^

Catalyst	BET surface (m^2^ g^−1^)	CO_2_ uptake (cm^3^ g^−1^ STP)		
		W	M	S
MgAl-LDO	167.89	5.24	40.81	0.36
CeMgAl-LDO	136.59	2.75	103.43	7.44
CeMgAl-LDO(P123)	144.94	4.03	137.87	12.99
SiO_2_@CeMgAl-LDO	342.11	4.27	129.08	13.05
SiO_2_@CeMgAl-LDO(P123)	358.71	3.66	145.62	13.59

a W, weak basic site; M, medium basic sites; S, strong basic sites, cm^3^ g^−1^ STP.

**Fig. 8:**
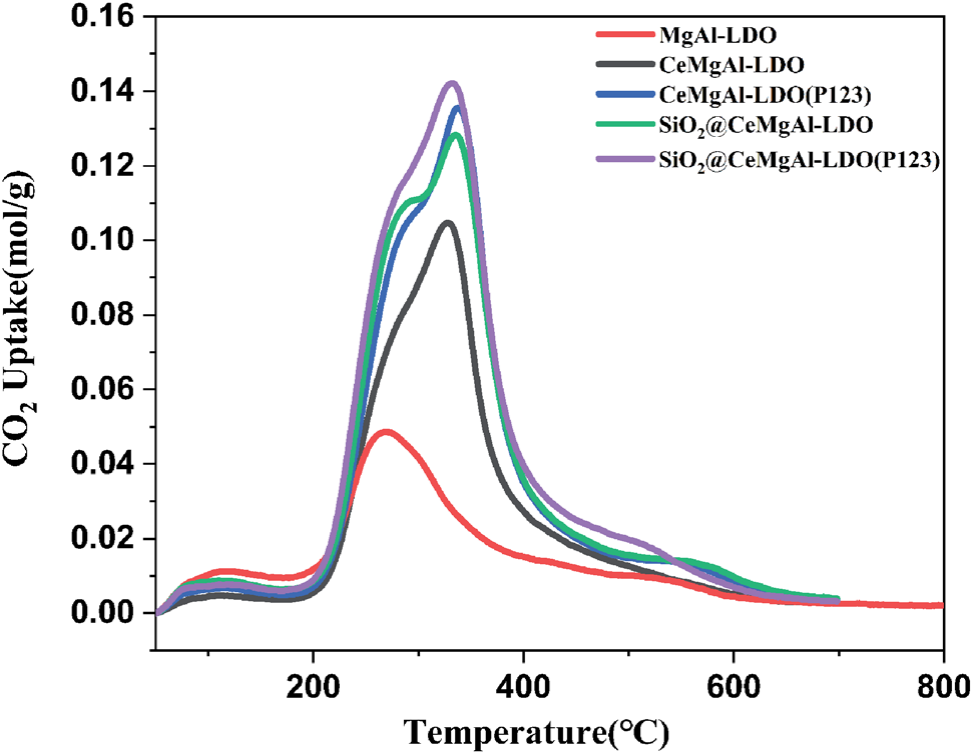
CO_2_-TPD profile of all as-prepared composite materials.

The CO_2_-TPD was used to analyze the effect of the strength and number of basic sites on the surfaces of the catalysts and the results were presented in Fig. 8 and Table 1. It could be seen that all the catalysts displayed CO_2_ desorption peaks at a similar temperature range of 200–400 °C and 500–600 °C. The low-temperature desorption peaks centered at 270 °C could be contributed to weakly CO_2_ chemisorbed by aluminum hydroxy and MgO.^37^ The peaks located at about 325 °C could be assigned to the medium basic sites derived from the oxygen vacancies formed during the doping of cerium in MgAl-LDO based composite. Combined with the results of specific surface area measurement, the core–shell structure has more complete stereoscopic structure and provides more alkaline sites at the same time. Moreover, the material with P123 template has the strongest total alkali content and strong alkali center strength.

### Catalytic synthesis of Propylene glycol methyl ether

Propylene glycol methyl ether (PGME) is used as an “alkahest” solvent in industrial and consumer product. It is generally produced by the reaction of propylene oxide (PO) with methanol, whose mechanism depends on the type of catalyst. As shown in Table 2, the epoxide ring of PO may open at the least sterically hindered position in basic catalysis, leading to the main formation of propylene glycol methyl ether (a).

**Table 2:** Catalytic activity for the synthesis of PGME on different catalysts

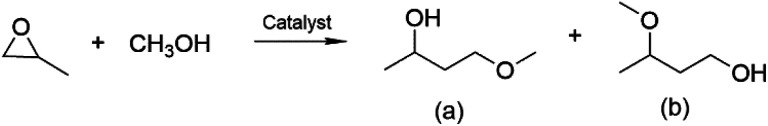						
Catalyst	Experimental conditions				Conversion of PO (%)	Selectivity of PGME (%)
	Catalyst (wt%)	Me/PO (mol mol^−1^)	*T* (°C)	Time (h)		
—	—	4	120	8	21.3	58.2
SiO_2_	1	4	120	8	23.8	74.2
Al_2_O_3_	1	4	120	8	60.3	70.6
Mg_3_Al-LDO	1	4	120	8	58.5	83.8
*^7^Al-MM^R^	3	8	60	6	98.7	61.2
*^9^12%KF/La_2_O_3_-SBA-15	1	5	125	4	93.0	91.0
*^40^ZIF-8	1.85	8	120	5	55.4	93.8
*^40^MAF-5	1.85	8	110	5	90.3	92.6
*^11^AM-4	1.8	5	110	5	88.4	92.3
*^6^2.9%ZIF-8/Nafen	1.6	5	120	5	84.1	86.3
Ce_0.33_Mg_2_Al_1_-LDO	2	4	120	8	96.7	89.6
Ce_0.33_Mg_2_Al_1_-LDO(P123)	2	4	120	8	97.6	89.8
Ce_1_Mg_3_Al_1_-LDO(P123)	2	4	120	8	97.8	86.3
SiO_2_@Ce_0.33_Mg_2_Al_1_-LDO	2	4	120	8	97.8	91.6
SiO_2_@Ce_0.33_Mg_2_A_1_-LDO(P123)	2	4	120	8	99.7	92.4
SiO_2_@Ce_1_Mg_3_A_1_-LDO(P123)	2	4	120	8	90.3	86.5
SiO_2_@Ce_0.3_Mg_3_Al_1_-LDO(P123)	2	4	120	8	94.6	90.4
SiO_2_@Ce_0.05_Mg_3_Al_1_-LDO(P123)	2	4	120	8	88.3	89.6

The mechanism of the reaction on different single component basic catalysts has been discussed.^38,39^ The moderate basic sites of Lewis-acid–Bronsted-base pairs and isolated surface O^2−^ ions were appropriate to dissociate methanol to methoxide and had the ability to abstract a proton from propylene oxide.

In the present work, the catalytic performance of the as-prepared core–shell composites was tested in the synthesis of propylene glycol methyl ether from methanol and PO. Table 2 shows that the conversion of PO in the presence of SiO_2_@Ce_0.33_Mg_2_Al_1_-LDO(P123) was 99.7%, a relatively high level among the prepared and reported catalysts. Although ZIF-8 has the best selectivity for propylene glycol monomethyl ether, it is difficult to apply ZIF-8 in the actual production process considering the stability and cost of the catalyst. The selectivity towards PGME was higher in the presence of SiO_2_@Ce_0.33_Mg_2_Al_1_-LDO(P123) (92.4%) than other heterogeneous catalysts. Probably, this phenomenon is related to the difference in the strength of the moderate basic sites. The activity of CeMgAl-LDO is greatly higher than those of the MgAl-LDO or pure Al_2_O_3_ sample, indicating that the introduction of Ce ions into the Mg–Al catalysts can significantly enhance the catalytic performance. The CeO_2_ grains were uniformly distributed on the LDO structure and interacted strongly with Mg–O–Al bonds. This strong interaction leads to the formation of coordinately unsaturated surface Ce^3+^ cations. Besides, the introduction of Ce cations in Mg–Al mixed oxides can modify the acid–base properties of the mixed oxides. Some studies have shown that the aggregation of CeO_2_ grains can weaken the catalytic performance of the catalyst. Therefore, the specific gravity of Ce in the composite should not be too large, and the agglomeration of CeO_2_ should be avoided. According to TEM analysis, when P123 was introduced, CeO_2_ was well dispersed, which was consistent with the phenomenon that the catalytic performance of P123 modified materials was significantly better than that of unmodified materials. According to the literature,^41,42^ the strength of basic sites determines the reaction rate and the isomer selectivity toward PGME. Our experimental data also point out that strong basic sites lead to a decrease in the selectivity of PGME. It is reported that a high strength of strong basic sites can lead to a strong stabilization of methoxide and propylene-like species which formed as intermediates on the surface of the solid and, in this way, hinder the process of reaction.^8^

The element ratio of the catalyst and the reaction conditions were also studied. In Table 2, we investigated the element ratio of the catalyst and found that SiO_2_@Ce_0.33_Mg_2_A_1_-LDO(P123) has the best catalytic activity. When the amount of Ce increased, although the conversion of PO did not decrease, the selectivity of PGME decreased significantly. In the TEM image analysis (Fig. S1†) of Ce_1_Mg_3_A_1_-LDO(P123), more complex lattice fringes and crystal structure can be found, and the agglomeration of CeO_2_ is serious. When the specific gravity of the crystal plane which plays the main catalytic role decreases, the selectivity will be affected negatively. Then in the exploration of reaction conditions, too long reaction time and high temperature will affect the selectivity of PGME. This is because that under a certain proportion or environmental conditions, PGME and PO will polymerize to form polypropylene glycol monomethyl ether with different degrees of polymerization, which will seriously affect the quality of the product. After single factor optimization (Fig. S3†), the conversion of PO reaches 99.7% and selectivity of PGME reaches 92.4% at a molar ration Me/PO 4 : 1 and 120 °C for 8 h *via* 1 wt% SiO_2_@Ce_0.33_Mg_2_A_1_-LDO(P123).

**Fig. 9:**
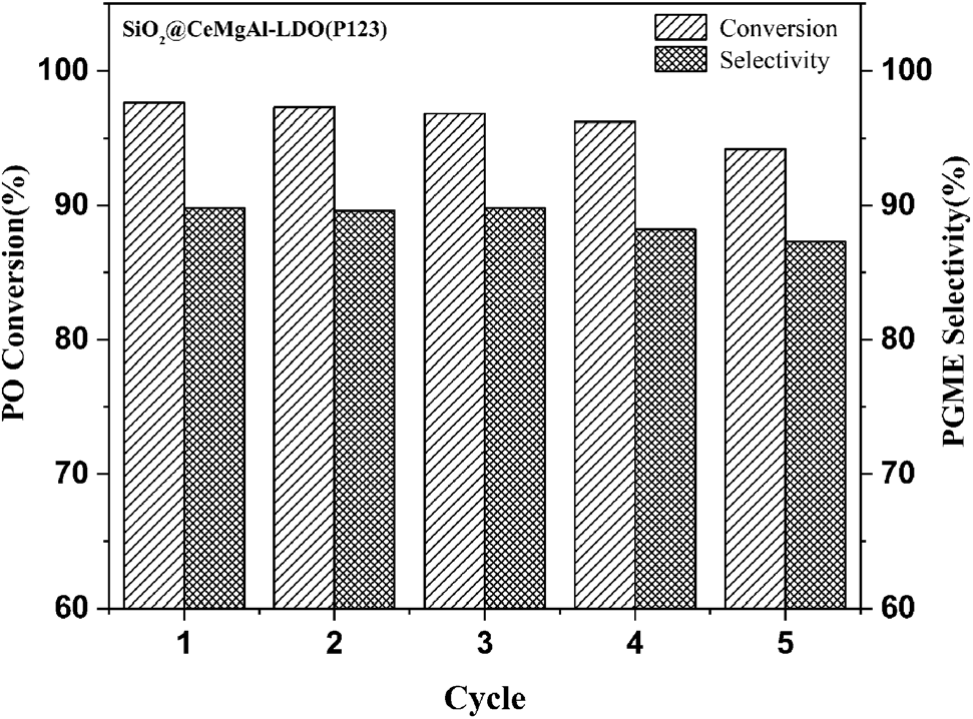
Recycling test in reaction between propylene oxide and methanol in the presence of SiO_2_@Ce_0.33_Mg_2_Al_1_-LDO(P123).

The catalyst was separated by filtration after the first test, washed with methanol, dried in air and activated at 550 °C for 6 h, and then reused for the next run under the same conditions. The results are depicted in Fig. 9. As shown in the figure, the catalytic activity of the reused catalyst was slightly affected at the fifth run, exhibiting >93.0% conversion and retaining 87.0% selectivity to PGME. These results imply that the catalyst can be efficiently recovered and recycled.

## Experimental section

### Materials

All chemicals employed in this study were analytical grade, from commercial sources. Magnesium nitrate hexahydrate (Mg(NO_3_)_2_·6H_2_O), cerium nitrate hexahydrate (Ce(NO_3_)_3_·6H_2_O), and aluminum nitrate hexahydrate (Al(NO_3_)_3_·9H_2_O) were purchased from Zhenxin Reagent Co., Ltd. Sodium hydroxide (NaOH), sodium carbonate (NaCO_3_), and tetraethyl orthosilicate (Si(OC_2_H_5_)_4_, TEOS) were purchased from sinopharm chemical reagent Co., Ltd. Epoxy propane (C_3_H_6_O), ethanol (C_2_H_5_OH), and methyl alcohol (CH_3_OH) were purchased from Shanghai Lingfeng Chemical Regent Co., Ltd. Poly(ethylene glycol)-*block*-poly(propylene glycol)-*block*-poly(ethylene glycol)(P123) was purchased from Aladdin.

### Preparation of SiO_2_ spheres

Spherical SiO_2_ was prepared according to the previous report.^43^ 13.7 ml TEOS, 15 ml ammonia, 30 ml deionized water and 50 ml ethanol were combined in a 250 ml eggplant flask. The white suspension was stirred vigorously at 25 °C for 18 h. The obtained solid was centrifuged for 5 min at 3000 rpm then washed with ethanol thoroughly followed by drying under vacuum overnight.

### Preparation of CeMgAl-LDH(P123) and SiO_2_@CeMgAl-LDH(P123)

The SiO_2_ was covered with P123-templated CeMgAl-LDH by *in situ* growth of the metal cations. Typically, SiO_2_ spheres (0.5 g) and Na_2_CO_3_ (20 mmol) were dispersed in distilled water by ultrasonication for 30 min. Another solution of Ce(NO_3_)_3_·6H_2_O (4 mmol), Mg(NO_3_)_2_·6H_2_O (24 mmol), Al(NO_3_)_3_·9H_2_O(12 mmol) were prepared in distilled water (100 ml, in the presence of 1.0 g P123), then were added in the above solution drop-by-drop at vigorous stirring at 40 °C for 18 h. During the reaction process, the pH was kept at 10 by the addition of 2 M NaOH. The acquired mixture was washed with ethanol and drying at 60 °C in a vacuum oven overnight. (SiO_2_@CeMgAl-LDH(P123)). To obtain the mixed oxides (SiO_2_@CeMgAl-LDO(P123)), the precursor was calcined at 550 °C for 6 h in a muffle furnace in air at a heating rate of 5 °C min^−1^. The synthetic steps of the composite materials were shown in Fig. 1. The pure LDH(CeMgAl-LDH(P123)) and LDO(CeMgAl-LDO(P123)) were prepared by same co-precipitation method without the addition of SiO_2._

### Characterizations

The powder X-ray diffraction patterns (XRD) measurements were performed on a X-ray diffractometer (Rigaku Co., Japan, D/max 2500VL/PC) using Cu Kα radiation (40 kV, 100 mA), and a 2*θ* angle ranging from 2° to 90°, respectively. The HRTEM images were obtained on JEM-2100, ZEOL-Japan in which the samples were prepared by dispersing the powdered samples in ethanol by sonication for 15 min and then dropdrying on a copper grid coated with carbon film. The thermogravimetric (TG) was carried out in the temperature range of 25 to 800 °C with a heating rate of 10 °C min^−1^ and air flow of 100 ml min^−1^ on a Diamond TG/DTA/DSC instrument (PerkinElmer, USA). The FT-IR spectra of the materials were recorded on Hyperion-2000 (Bruker, Germany). The specific surface areas were calculated using the Brunauer–Emmett–Teller (BET) method based on the N_2_ adsorption isotherms (ASAP 2020, Micrometrics, USA).

### Catalytic tests

Catalytic properties of as-prepared materials were tested in the synthesis of 1-methoxy-2-propanol (PGME) from methanol and propylene oxide (PO) at 120 °C. The catalysts were activated at 150 °C for 2 h before the reaction to remove adsorbed water. The reaction was carried out in a stainless steel autoclave reactor with an inner volume of 500 ml. The reaction was typically performed using 0.5 mol of PO, 2 mol of MeOH and catalyst (0.93 g, 1 wt%). After 8 h of reaction, the reactor was cooled down to room temperature. The products were analyzed using GC (Thermo Fisher Trace 1300) with a FID detector equipped with PEG-20M column.

## Conclusions

In this work, core–shell Ce-modified mixed metal oxides derived from P123-template layered double hydroxides were studied in synthesis of propylene glycol methyl ether. SiO_2_@Ce_0.33_Mg_2_Al_1_-LDO(P123) displayed the highest catalytic activity, which could realize 99.7% conversion of propylene oxide and at 120 °C. Introduction of cerium and structural regulation of P123 template make more active sites appear, which can improve the selectivity of propylene glycol monomethyl ether. On the other hand, the formation of core–shell structure improves the dispersion of the catalyst, and overcomes the disadvantage of easy agglomeration of hydrotalcite. The coprecipitation method ensures the relatively stable structure of the core and shell, and improves the service life of the catalyst. This work provides a new perspective on core–shell layered double hydroxide and contributes to the design of high performance heterogeneous catalyst.

## Conflicts of interest

There are no conflicts to declare.

## Supplementary Material

RA-011-D1RA00227A-s001
