# Supplementary material for: Synthesis of core–shell Ce-modified mixed metal oxides derived from P123-templated layered double hydroxides
Source: RSC Adv. 2021 Feb 23;11(14):8375–83. doi: 10.1039/d1ra00227a (PMC8695133; doi:10.1039/d1ra00227a)
Supplement: RA-011-D1RA00227A-s001 [file RA-011-D1RA00227A-s001.pdf]

Electronic Supplementary Material (ESI) for RSC Advances.
This journal is © The Royal Society of Chemistry 2021

Supplementary Information

Synthesis of Core-Shell Ce-modified Mixed Metal Oxides Derived from P123-Templated Layered Double Hydroxides

Kaijun Wang, [a] Qifan Mao, [a] Weimin Fei,[a] Lingxin Kong,[a] Xiaoyan Cao, [a] and Zhenggui Gu*[a]

[a] Jiangsu Provincial Key Laboratory of Materials Cycling and Pollution Control, Nanjing Normal Univesity, Nanjing, Jiangsu 210023 China.

*Corresponding authors: Zhenggui Gu

Email:07160@njnu.edu.cn

Supporting Figures

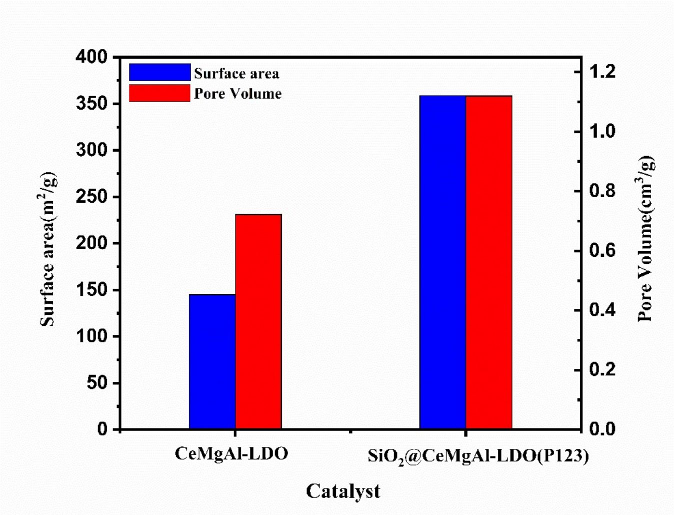

Figure S1 Surface area and Pore Volume of samples.

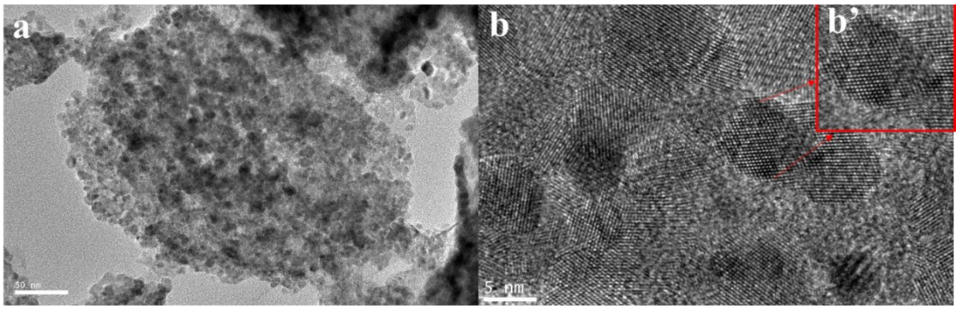

Figure S2 TEM images of $Ce_1Mg_3Al_1$-LDO.

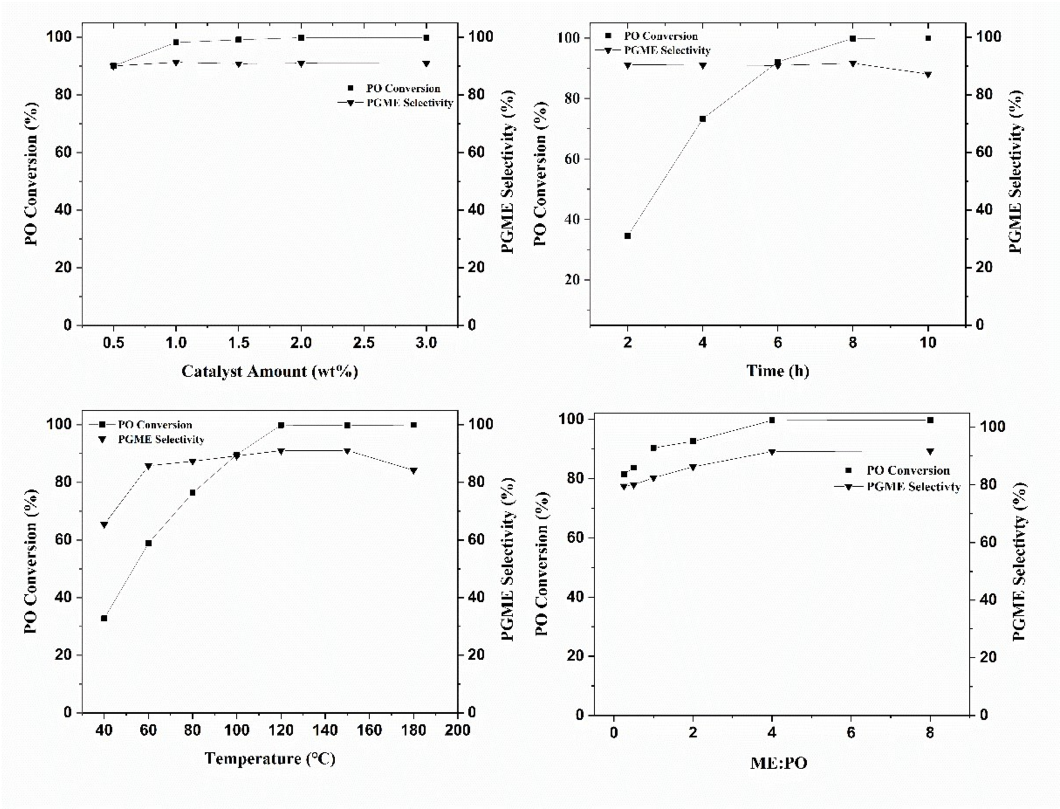

Figure S3 Single factor optimization of the reaction.
